# Chitosan-Lignin Cryogel Beads for the Adsorption of Diclofenac in Water

**DOI:** 10.1021/acs.biomac.6c00420

**Published:** 2026-07-10

**Authors:** Ana Zidar, Alejandro Lorente, Daniel Kutifa, Stephanie Spahr, Mohsen Adeli, Rainer Haag, Olaf Wagner

**Affiliations:** † Institute of Chemistry and Biochemistry, 9166 Free University of Berlin, Berlin 14195, Germany; ‡ Department of Ecohydrology and Biogeochemistry, 28401 Leibniz Institute of Freshwater Ecology and Inland Fisheries (IGB), Berlin 12587, Germany; § Department of Organic Chemistry, Faculty of Chemistry, Lorestan University, Khorramabad 68141-54316, Iran

## Abstract

The production, use, and disposal of pharmaceuticals contribute to widespread pollution of the aquatic environment. Conventional wastewater treatment plants are not designed to completely remove bioactive pharmaceuticals such as diclofenac, posing risks to aquatic ecosystems and drinking water sources. To address this challenge, we report the production of biogenic adsorbent beads for enhanced removal of anionic contaminants from water. The beads consist of glutaraldehyde-cross-linked chitosan (or quaternary amine-functionalized chitosan) combined with lignin, and a macroporous structure was introduced via freeze-drying. The resulting cryogel beads were characterized and benchmarked against two commercially available adsorbents, an activated carbon and an ion-exchange resin. Compared to these reference materials, the presented cryogel beads exhibit significantly faster diclofenac removal kinetics and a higher adsorption capacity. Furthermore, upon treatment with a brine solution, the cryogel beads can desorb diclofenac and show good performance over five adsorption–desorption cycles. The present study demonstrates the potential of biopolymer cryogel beads as sustainable materials for water treatment.

## Graphical abstract

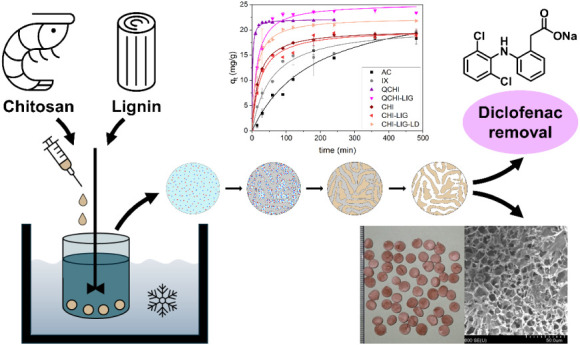

## 1 Introduction

Water resources are increasingly under threat due to depletion and contamination arising from industrial discharges, agricultural runoff containing fertilizers and pesticides, and inadequately treated municipal wastewater.^1^ Of particular concern are persistent and mobile anthropogenic organic substances that pose a risk to freshwater quality, as they can pass through natural barriers, such as soil filtration, and conventional wastewater treatment processes and thus accumulate in the water cycle.^2^ Among these substances, pharmaceuticals pose a growing risk to aquatic organisms and ecosystems due to their biological activity even at low concentrations.^3^ Pharmaceuticals can enter wastewater, for example, through improper disposal, excretion after use in humans and animals, or release from industrial sites.^4,5^ Effluent from pharmaceutical manufacturing facilities is often highly contaminated and requires improved and effective treatment.

An example of a pharmaceutical pollutant widely detected in the aquatic environment is diclofenac (DF), an anionic and highly mobile organic compound. DF is a nonsteroidal anti-inflammatory drug, used for treatment of pain and inflammation. It has been detected in groundwater at a concentration of 590 ng/L and drinking water at 35 ng/L.^2,3,6^ DF is known to be degradable by UV light, but exhibits low biodegradability and only moderate removal by wastewater treatment plants, so additional treatment is necessary to reduce its release into the aquatic environment.^6–8^

Among the available water treatment methods, adsorption is a promising approach for the removal of persistent organic contaminants, due to its operational simplicity, absence of toxic transformation byproducts, and high removal efficiency.^9,10^ Common commercially available adsorbents are activated carbon (AC) or ion-exchange resin (IX). AC is commonly derived from nonrenewable resources such as coal and used for the removal of heavy metals, dyes, pharmaceuticals, and numerous trace organic contaminants. However, AC is not effective for the removal of mobile compounds (e.g., short-chain per- and polyfluoroalkyl substances (PFAS) or other small organic polar or ionizable compounds).^11–15^ Commercial ion-exchange resins are typically cross-linked synthetic polystyrene or polyacryl resin beads.^16^ The selectivity and adsorption properties of the resins can be tuned through modification of grafted functional groups.^17^ However, IX may release microplastics during use or after disposal.^16^

These limitations highlight the need for the development of alternative adsorbents based on biogenic materials. These materials have the potential to improve the removal of persistent and mobile organic contaminants that exhibit rapid breakthrough in conventional AC (waste) water treatment systems, while reducing reliance on fossil resources and preventing additional environmental contamination by microplastics. Among biogenic materials, chitosan and lignin are of particular interest as they can be derived from waste streams and exhibit favorable physicochemical properties.

Chitosan, which can be derived from chitin, a material from seafood waste streams, has been widely studied in recent years and shown to be an effective precursor for adsorbents capable of removing heavy metals, dyes, pharmaceuticals, oils, and pesticides,^18^ or even as an antimicrobial agent due to its abundant free amino and hydroxyl functional groups.^19^

Lignin, the second most abundant biopolymer after cellulose, is another waste-derived biomaterial, originating from the paper industry.^20^ Beyond its conventional use in energy recovery through combustion, lignin is being explored as a sustainable feedstock for chemicals and materials, for instance as an adsorbent material for the removal of dyes and heavy metals.^21^

Formulation of chitosan and lignin into beads, microbeads, films, nanofibers, composites, resins and sponges for different applications has been reported in literature.^22,23^ Of these forms, porous beads- similar to commercially available IX- are particularly well-suited for use in adsorption applications due to their high specific surface area. Compared to powders, bead- shaped adsorbents offer several advantages, particularly for filter systems in water treatment, for example to allow better flow-through performance with lower pressure drop, reduced clogging and channeling, easier handling, simpler packing and refilling of cartridges, and efficient backwashing.^24,25^

Our research focused on tailoring a porous bead synthesis procedure using two widely studied biopolymers, chitosan and lignin, to develop biogenic adsorbent materials for the removal of organic contaminants from water. Cryogelation was used in the synthesis procedure to increase the surface area of the materials. In this work, five variations of adsorbent beads were tested and compared against commercial AC and IX. Beads were synthesized using either native chitosan or chitosan modified with quaternary ammonium groups to investigate the effect of the high content of cationic binding sites. In three compositions, lignin was used as a complementary biopolymer filler, and one formulation was prepared at half concentration to increase the pore-to-polymer-matrix ratio, thereby enhancing porosity. The adsorption performance of the prepared materials was assessed using diclofenac as a model pollutant. Four of the five synthesized cryogel bead materials exhibited superior diclofenac uptake compared to conventional adsorbents.

## 2 Experimental Section

### 2.1 Materials

Chitosan (90/10/1A, 90% deacetylation degree, viscosity of 1% solution in 1% acetic acid = 10 mPas, molecular weight 20–40 kDa, <1% ash content) was purchased from BioLog Heppe GmbH. Lignin (UPM BioPiva 100, a purified, softwood kraft lignin, 65% solid content at 105 °C, molecular weight 5 kDa, <2% ash content at 700 °C) was purchased from UPM Biochemicals. Glycidyltrimethylammonium chloride (GTMAC, 80%) and diclofenac sodium salt (DF, 98%) were purchased from TCI. Glutaraldehyde (50%), sodium borohydride (98%), hexane (>95%), acetone (99.8%), sodium hydroxide – pellets, sodium chloride (≥99.5%), and sodium hydrogen carbonate (>99.7%) were purchased from Thermo Fisher Scientific. Ethanol was purchased from Berkel AHK Alkoholhandel GmbH and Co KG. Activated carbon FILTRASORB 400 was purchased from Chemviron. Ion-exchange resin Lewatit VP OC 1065 was acquired from Lanxess GmbH. Water was ultrapurified by deionization and filtration before use.

### 2.2 Methods

#### 2.2.1 Synthesis of Quaternized Chitosan (Quaternary Amine-Functionalized Chitosan)

Chitosan (10.0 g) was dispersed in 380 mL aqueous acetic acid (1% v/v).^26^ The solution was stirred for 24 h at 60 °C until chitosan was fully dissolved. Quaternizing agent GTMAC (37.6 g) was dissolved in water (20 mL) and added to the reaction mixture, which was stirred for further 18 h at 60 °C. The reaction mixture was cooled to room temperature, then precipitated in an acetone/ethanol mixture (10:1). The precipitate was filtered and redissolved in water at 60 °C, then cooled and precipitated again in acetone. Quaternized chitosan precipitated as white solids, which were collected and dried in the oven at 60 °C.

#### 2.2.2 Synthesis of Cryogel Beads

Chitosan or quaternized chitosan solutions were prepared by dissolving the polymer in aqueous acetic acid (1% v/v) or water at 60 °C and cooled to 0 °C. Optionally, lignin was homogeneously dispersed in the polymer solution (the detailed feed composition for each composite can be found in Table S1). The cross-linking agent GA was added to prepare polymer solutions of 4 wt % (2 wt % for CHI-LIG-LD), with GA added in 1:10 weight ratio relative to the polymer and stirred vigorously until homogenization of the mixture. The polymer/GA or polymer/GA/lignin solutions were then injected through a syringe with a 1.2 mm inner-diameter needle into hexane at −30 °C from a height of 10 cm. The resulting beads were transferred to a precooled beaker and kept in a freezer overnight to ensure completion of the cross-linking reaction under cryogelation conditions. The frozen beads were freeze-dried to obtain anhydrous cross-linked chitosan beads. In the case of QCHI and CHI, the beads were washed for 4 h in a reductive solution consisting of 2 g NaBH_4_, 2.1 g NaHCO_3_, and 0.43 g NaOH dissolved in 1000 mL of water. The beads were washed with water until the wash returned to neutral pH, then frozen and lyophilized.

*Fourier transform infrared spectroscopy* (FTIR) measurements were performed using a FT/IR-4100 spectrometer (Jasco, Hachioji, Japan) equipped with an Attenuated Total Reflection unit.

*Nuclear magnetic resonance* (NMR) ^1^H spectra were recorded using an ECX 600 MHz spectrometer (Bruker, Karlsruhe, Germany). The NMR spectra of chitosan were measured in D_2_O with the addition of two drops of deuterium chloride 20% solution in D_2_O and of quaternized chitosan in D_2_O.

*Scanning electron microscopy (SEM)* was performed with a field emission scanning electron microscope (FE-SEM, Hitachi SU8030, Japan) at an accelerating voltage of 15 kV, and a current of 10 μA. Briefly, the samples were first lyophilized in a freeze-dryer. When the samples were dried until constant weight was achieved, they were sputtered with 5 nm gold by using a sputter coater (Emscope SC 500, Quorum Technologies, UK) for 30 s under argon atmosphere at a current of 30 mA and a pressure of 10^–1^ Torr (1.3 mbar), before SEM imaging.

*Mean bead diameters* of cryogel beads were determined using representative images of the beads and ImageJ software.

*Swelling degree* of cryogel beads was determined by immersing the beads into water and measuring their mass before swelling and after 24 h in water. SD was then calculated according to eq 1, where W_d_ is the dry weight of the cryogel bead and W_s_ is the weight of the swollen bead.

1 $$swelling\;degree\;\left(\%\right)=\frac{W_{s}-W_{d}}{W_{d}}\times 100$$

Three parallel samples of each bead type were chosen, and the mean swelling degree of the three samples was taken as the final swelling degree.

*Bulk density* of cryogel beads was determined by two methods. In the first method, bulk density was determined by measuring the diameter of the beads and their weight and then calculated according to eq 2, where W_d_ is the dry weight of the cryogel bead and d is the bead diameter.

2 $$density=W_{d}/\left(\frac{4}{3}\times \pi \times {\left(\frac{d}{2}\right)}^{3}\right)$$

Three parallel samples of each bead type were chosen, and the mean density of the three samples was taken as the final density.

*Mercury intrusion porosimetry* was used to determine mode pore diameter, pore size distribution, and bulk density, using a MicroActive AutoPore V porosimeter.

*Liquid chromatography electrospray ionization tandem mass spectrometry (LC-ESI-MS/MS)* was used to determine DF concentrations for kinetic and thermodynamic adsorption studies. Measurements were carried out using an Agilent 1290 Infinity II LC system coupled to an Agilent 6470 triple quadrupole mass spectrometer. Aqueous samples were analyzed using a XBridge BEH C18 column (5 cm × 2.1 mm, 3.5 μm, WATERS) equipped with a XBridge BEH C18 VanGuard cartridge (5 mm × 2.1 mm, 3.5 μm, WATERS). The eluent mixture consisted of LC-MS grade H_2_O (solvent A, LiChrosolv, Sigma) and LC-MS grade methanol (solvent B, *≥*99.95%, Rotisolv, Roth), each acidified with 0.1% formic acid (Fluka). The eluent gradient increased linearly from 10% B to 95% B in 15 min and was kept constant at 95% B for 1 min at a flow rate of 200 μL/min. Electrospray ionization (ESI) was used in positive mode and DF was measured in multiple reaction monitoring (MRM) with a precursor mass [M + H]^+^ of 296.0 *m*/*z* and three product masses [M + H]^+^ of 214.1 *m*/*z* (quantifier), 215.0 *m*/*z* (qualifier), and 250.0 *m*/*z* (qualifier). DF quantification was performed using external calibration standards from 1–500 ng/mL and the Agilent MassHunter Workstation software. The lowest calibration standard with a signal-to-noise ratio ≥10 was defined as the limit of quantification.

*UV–vis spectroscopy* was used to determine DF concentrations for the desorption study. UV–vis spectroscopy experiments were performed with an Agilent Cary 8454 UV–Vis spectrophotometer and quartz cuvette at 276 nm. Calibration curves for DF in deionized water and 0.1 M NaCl were established by recording the UV–vis absorption over a range of concentrations.

*Elemental analysis* was performed using an Elementar Vario EL machine equipped with three columns and heat conductivity meter.

*Adsorption kinetic experiments* were performed using 40 mg/L DF solution, prepared by dissolving diclofenac sodium in deionized water. Kinetic experiments were performed in 50 mL flasks, containing 40 mg of adsorbent materials and 25 mL of 40 mg/L DF solution. The flasks were agitated on a horizontal shaker at room temperature for defined times, ranging from 10 to 480 min prior to sampling. The initial pH of the DF solution was 6.3, and it changed to 6.6, 4.6, 5.6, 4.6, and 4.6 over 480 min for QCHI, QCHI-LIG, CHI, CHI-LIG, and CHI-LIG-LD, respectively. The kinetic experiment was performed in triplicate for each material.

The parameters describing the adsorption kinetics of DF onto the adsorbents were estimated via nonlinear analysis of the data using eq 4 for the pseudo-first order kinetic model and eq 5 for the pseudo-second order kinetic model.^27–29^ The quality of fit of the experimental kinetics data was evaluated using the coefficient of determination (R^2^).

The adsorption capacity at time t, q_t_ (mg/g), was calculated according to eq 3:

3 $$q_{t}=\frac{C_{0}-C_{t}}{m}V$$

where C_0_ (mg/L) is the initial measured concentration of DF, C_t_ (mg/L) is the concentration of DF at time t, V (L) represents the volume of the DF solution, and m (g) represents the dry weight of adsorbent.

4 $$q_{t}=q_{e}\left(1-e^{-k_{1}t}\right)$$

5 $$q_{t}=\frac{k_{2}q_{e}^{2}t}{1+q_{e}k_{2}t}$$

qe (mg/g) is adsorption capacity at equilibrium, k_1_ (1/min) and k_2_ (g/(mg min)) are kinetic rate constants.

*Adsorption isotherm experiments* were utilized to study the equilibrium of DF adsorption on the adsorbents. The adsorption isotherms were obtained at room temperature. DF solutions were prepared by dissolving diclofenac sodium in deionized water. The batch tests were conducted by adding 10 mg of cryogel beads to 100 mL flasks containing 50 mL of DF solutions of varying concentrations (10, 20, 40, 70, 115, 160, 200, and 250 mg/L). Samples were agitated on a horizontal shaker until reaching equilibrium. The time of equilibrium was based on preliminary tests and was 48 h for IX, QCHI, QCHI-LIG, CHI, CHI-LIG, and CHI-LIG-LD, and 72 h for AC. The initial and final pH values of each measurement can be found in Table S2. The experiment was performed in triplicate for each material.

The adsorption isotherm data were analyzed using the Langmuir and Freundlich models to evaluate the adsorption capacity and surface heterogeneity of the materials.^30,31^

The adsorption isotherm data were analyzed using both the Langmuir (eq 6) and Freundlich (eq 7) models to evaluate the adsorption capacity and surface heterogeneity of the materials.

6 $$q_{e}=\frac{q_{max}K_{L}C_{e}}{1+K_{L}C_{e}}$$

qe (mg/g) is the adsorption capacity at equilibrium; Ce (mg/L) is the equilibrium concentration; qmax (mg/g) is the maximum adsorption capacity.

7 $$qe=K_{F}C_{e}^{1/n}$$

K_F_ ((mg/g)/(mg/L)^1/*n*^) is the Freundlich isotherm constant related to adsorption capacity and 1/*n* is a constant related to adsorption intensity.^30,31^

#### 2.2.3 Desorption Experiment

QCHI-LIG cryogel beads were loaded with 25 mL of 40 mg/L DF solution for 24 h. After 24 h, the DF concentration was measured in the solution to determine the amount of DF adsorbed. Then the beads were filtered and introduced into 50 mL of 0.1 M NaCl and shaken for 1 h. The DF concentration of the regeneration solution was measured to determine the amount of DF desorbed. The beads were then washed with deionized water. This cycle was repeated 5 times. The adsorption percentage was determined by eq 8

8 $$adsorption=100\%\times \left(1-\frac{C_{e}}{C_{0}}\right)$$

C_e_ (mg/L) is the equilibrium concentration and C_0_ (mg/L) is the initial concentration. The detailed calculation of the desorption percentages can be found in the Supporting Information (eq S1).

## 3 Results and Discussion

The adsorption mechanism of chitosan for anionic pollutants relies mainly on ionic interactions. At low pH its free amino groups become protonated (−NH_3_^+^), enabling intermolecular electrostatic attractions with DF.^32^ However, these amino groups have a reversible protonation equilibrium: at pH 6–7, chitosan is only partially protonated, limiting the number of available interaction sites. Grafting of quaternary ammonium groups onto the material should introduce pH-independent positive surface charges and stable adsorption properties.^26,33–37^ Quaternary ammonium groups were introduced into the chitosan backbone by grafting GTMAC onto the amino groups of chitosan, yielding quaternized chitosan (Figure 1a). The ring-opening reaction of the GTMAC epoxide can be performed under acidic or basic conditions.^26,38^ In this study, an acid-catalyzed reaction was used. For chitosan to dissolve, its amino groups must be protonated, which occurs partially at around pH = 6.5.^39^ Using a weak acid ensures that not all amino groups are functionalized with quaternary amine groups, and some remain available for further cross-linking reactions. Quaternized chitosan was characterized by FTIR and ^1^H NMR spectroscopy. A new band in the FTIR spectra at 1479 cm^–1^ was attributed to the C–H bending of the trimethylammonium group of the grafted GTMAC groups (Figure 1b). The degree of quaternization was determined using ^1^H NMR spectra via the peak area ratio of protons in the −N­(CH_3_)_3_ group to the H-3,4,5,6,6′ on the backbone of chitosan.^19^ The degree of quaternization was 87% (Figure 1c). The detailed calculation of the degree of quaternization can be found in the Supporting Information Figure S1 and eq S2.

**Figure 1:**
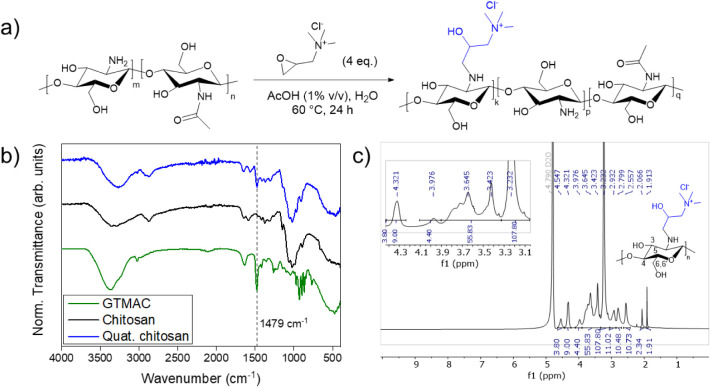
(a) Scheme for the synthesis of quaternized chitosan. b) IR spectra of glycidyltrimethylammonium chloride (GTMAC), chitosan, and quaternized chitosan. c) ^1^H NMR (600 MHz, D_2_O) spectra of quaternized chitosan.

There is an extensive list of reports addressing the formation of chitosan-based beads.^40^ Studies have shown that, compared with powder chitosan, which exhibits poor physicochemical characteristics, such as low surface area and porosity, physically modified chitosan forms demonstrate better adsorption performance.^41,42^ Formulating chitosan into gel beads may be an effective method to improve diffusion properties.^43^ We took inspiration from a strategy that has been previously explored for the synthesis of alginate-based cryogel beads.^44^ Cryogelation is a technique used to increase the surface area of materials by creating porous structures. In short, a polymer is dissolved in a solvent that is subsequently frozen, the formation of solid crystals develops a macroporous structure, which remains after sublimation of the solvent.^45^ To assess the effects of chitosan quaternization and lignin incorporation on DF adsorption, five types of beads were synthesized using either native or GTMAC-quaternized chitosan. Cryogel beads composed of quaternized chitosan (QCHI) and quaternized chitosan blended with lignin (QCHI-LIG) were prepared alongside unmodified chitosan (CHI) and chitosan blended with lignin (CHI-LIG). Additionally, a formulation with chitosan-lignin at half the total polymer concentration (CHI-LIG-LD) was synthesized to examine how reduced bead density and the potentially increased surface area would affect adsorption and bead stability. Lignin was incorporated into three of the cryogel bead formulations as a filler to evaluate its influence on adsorption performance and bead stability, and to investigate whether its aromatic structure could introduce additional interaction mechanisms, such as π–π stacking, with DF.^41^ Cross-linking of chitosan with GA resulted in the formation of imine bonds (Figure 2a). To avoid the reversibility of the amine-imine equilibrium and hydrolysis of the cross-linked polymeric backbone, the beads were treated with a reducing agent. Nonreduced beads, without lignin component, rapidly dissolved in deionized water, but they remained stable after treatment in a 2 wt % aqueous NaBH_4_ reduction bath. However, under the basic conditions of the reduction bath (pH = 10), lignin-containing beads exhibited lignin dissolution, as evidenced by brown color of the supernatant. In contrast, without reduction, the lignin-containing beads remained stable in water, and no lignin leaching was observed over a period of several months, during adsorption experiments or regeneration cycles. The introduction of lignin into the bead structure may provide sufficient intermolecular interactions to maintain structural integrity and to prevent bead disintegration arising from the reversible imine-amine equilibrium. Elimination of the reduction step is advantageous, since during the reduction step, the beads are soft and can easily be broken into smaller pieces under mechanical stress. Overall, lignin incorporation improves structural integrity and simplifies the synthesis process.

**Figure 2:**
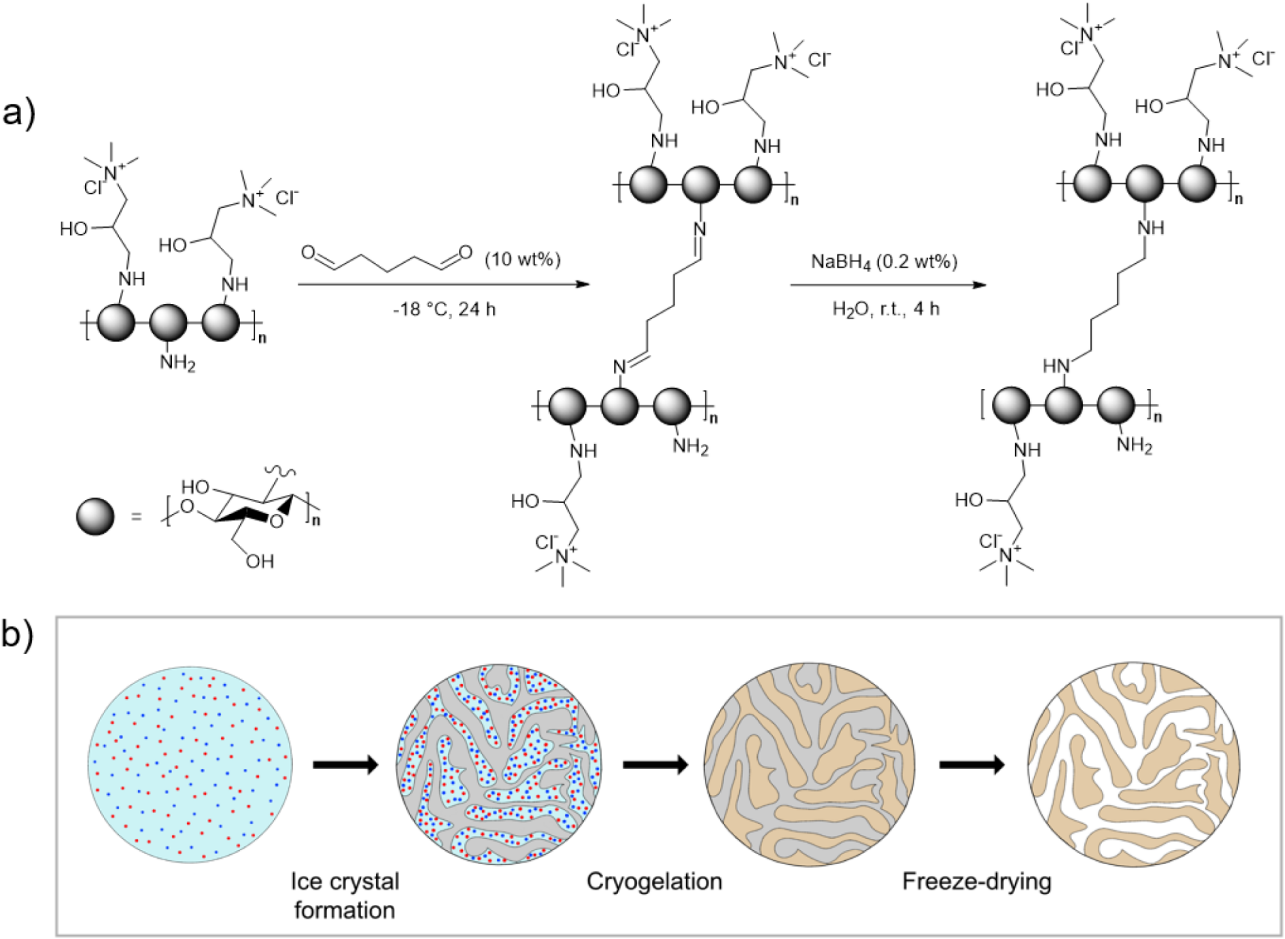
(a) Schematic representation of the cross-linking of functionalized chitosan by glutaraldehyde. b) Illustration depicting the formation and pore development mechanisms of cryogel beads.

In bead formation via a dropping method, the final size of the beads depends predominantly on the diameter of the nozzle.^46^ In this study, needles with an inner diameter of 1.2 mm were used. A smaller nozzle diameter to obtain smaller beads was not possible due to the high viscosity of the polymer solution. Photographic images of the cryogel beads confirm the formation of spherical beads, regardless of their composition. The bead size was analyzed by ImageJ software, indicating median diameters ranging from 3.85 mm to 6.69 mm (Figure 4b and Table S3). Beads containing lignin exhibited a characteristic brown coloration compared to their lignin-free counterparts (Figure 3a).

**Figure 3:**
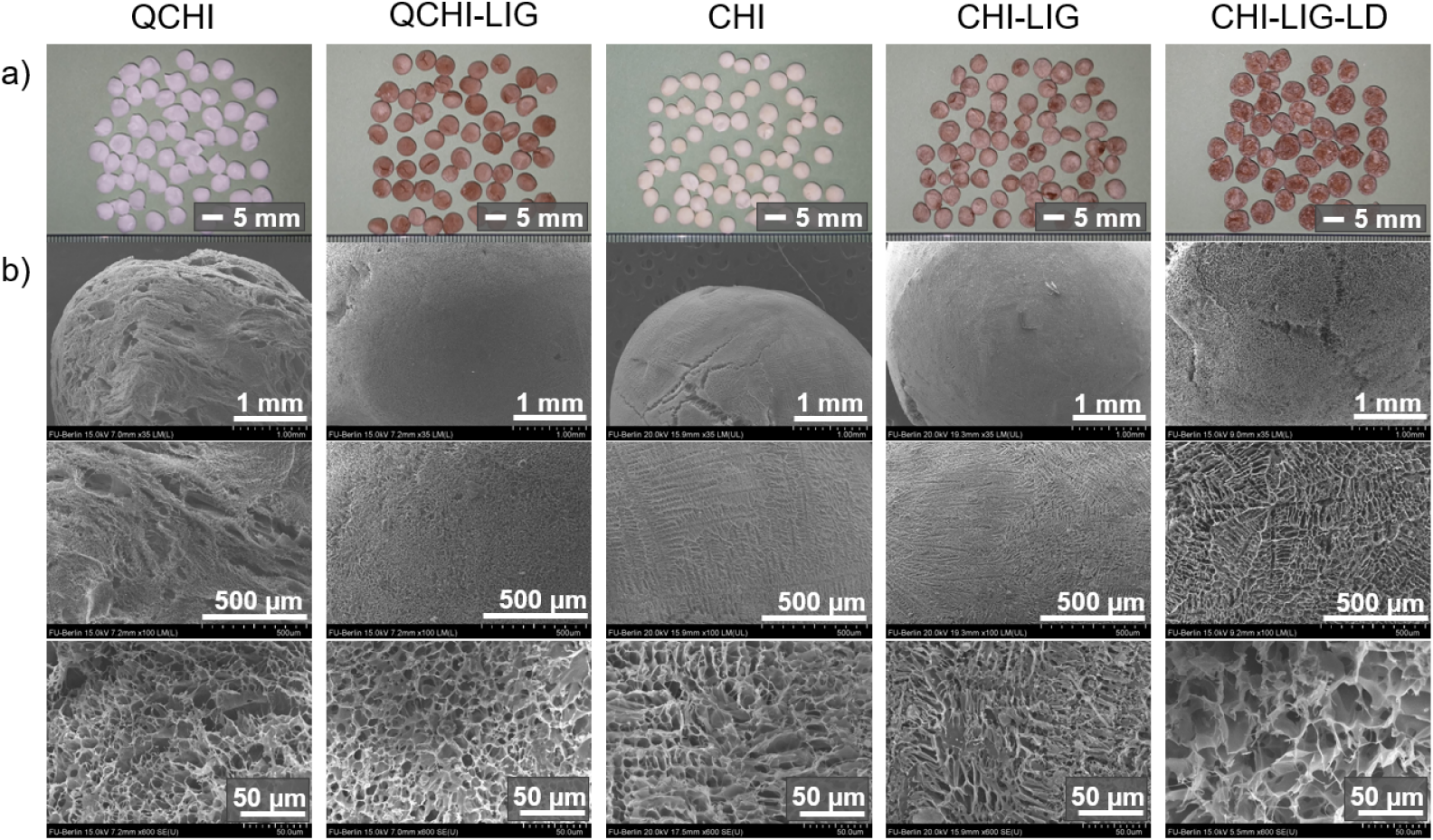
(a) Photograph images of cryogel beads with different compositions with mm scale. b) Scanning electron microscope images of the cryogel beads at different magnifications (35, 100, and 600, respectively).

The cryogel synthesis followed a sequence of steps in which freezing of the solvent (water) and subsequent sublimation rendered a macroporous structure in the resulting matrix (Figure 2b). The porous structure of cryogel beads was investigated using SEM (Figure 3b). SEM images at different magnifications indicated spherical morphology of the beads. At higher magnifications, a porous structure was recognized.

The swelling degree of all cryogel bead variants was determined (Figure 4a). Introduction of quaternary ammonium groups enhanced the hydrophilicity of the cryogels, resulting in higher swelling for QCHI and QCHI-LIG compared to their nonquaternized counterparts. In contrast, the incorporation of lignin particles reduced water uptake, as evidenced by the lower swelling degrees observed for QCHI-LIG versus QCHI and CHI-LIG versus CHI. Additionally, the increased porosity and reduced density of the CHI-LIG-LD beads led to an increase in water uptake relative to CHI-LIG.

**Figure 4:**
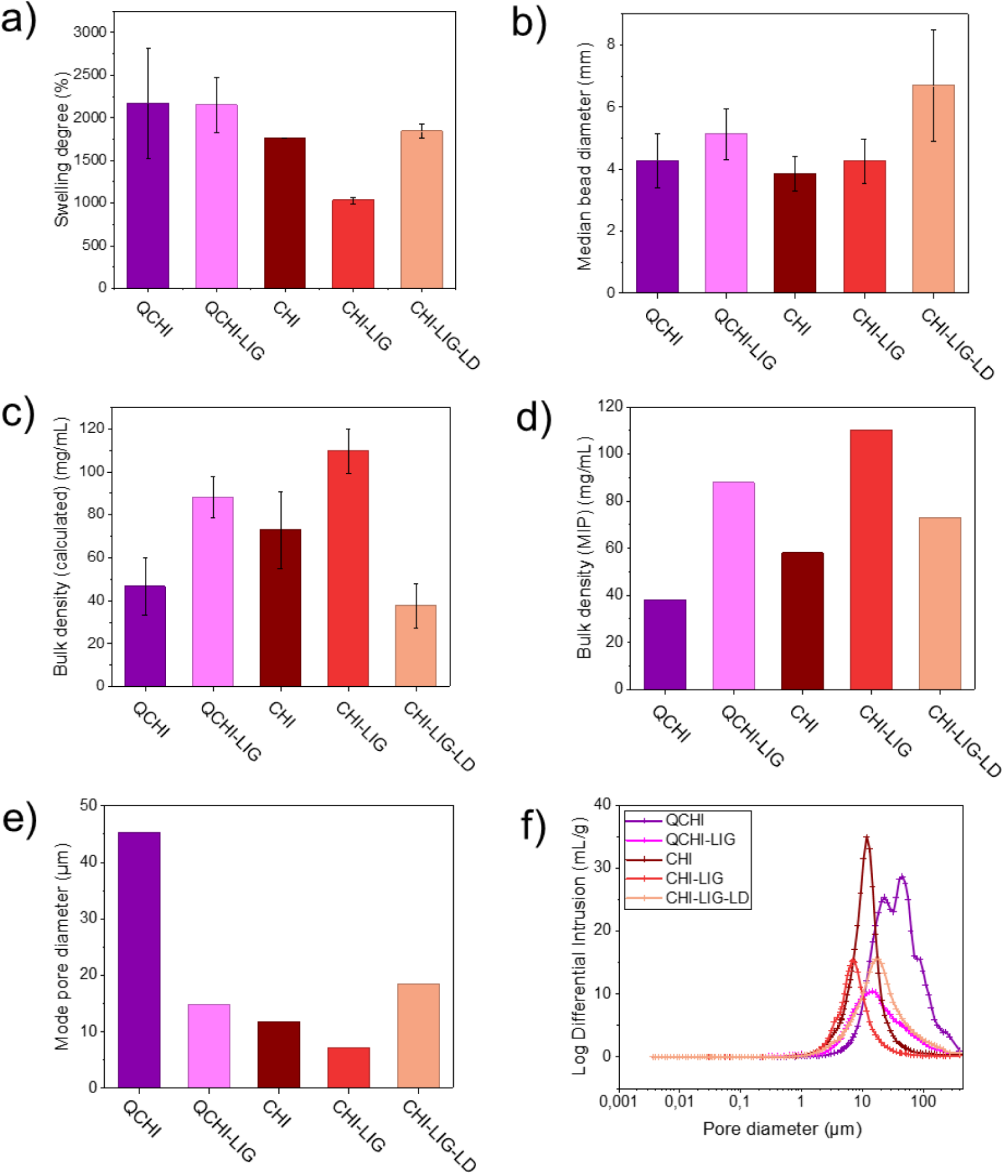
(a) Swelling degrees of cryogel beads. b) Mean bead diameters, determined from scanning electron microscopy images using ImageJ software. c) Bulk densities of cryogel beads, determined from bead weight and diameter. d) Bulk densities of cryogel beads, determined by mercury intrusion porosimetry (MIP). e) Mode pore diameters, determined by MIP. f) Pore diameter distributions, determined by MIP.

The bulk density of the materials was determined using two methods. In the first method, the bulk density was calculated from bead diameter and weight (Figure 4c). In the second method, the bulk density was determined using mercury intrusion porosimetry (Figure 4d). The results from both methods show good agreement. Comparison of lignin-containing materials to their lignin-free counterparts indicates that the addition of lignin increased bulk density. The bulk density was slightly higher for the nonquaternized materials compared to the quaternized materials. As expected, the lower polymer concentration in the production process of CHI-LIG-LD led to a decrease in bulk density compared to CHI-LIG.

Mode (most common) pore diameter values determined by mercury intrusion porosimetry were 45.20 μm, 14.72 μm, 11.76 μm, 7.06 μm, and 18.41 μm for QCHI, QCHI-LIG, CHI, CHI-LIG, and CHI-LIG-LD, respectively. The pore size distribution can be seen in Figure 4f. The incorporation of lignin led to a reduction in pore size in cryogel beads of otherwise comparable composition. Specifically, QCHI-LIG and CHI-LIG exhibited mean pore diameters of 14.72 and 7.06 μm, respectively, which are smaller than those of their lignin-free counterparts, QCHI (45.20 μm) and CHI (11.76 μm). This observation is consistent with the enhanced structural stability of lignin-containing beads. Lignin is a highly functionalized aromatic biopolymer containing phenolic hydroxyl, aliphatic hydroxyl, and carboxyl groups, while chitosan provides amine and hydroxyl functionalities. These groups can interact through hydrogen bonding, electrostatic interactions, and π–π interactions, which facilitate additional physical cross-linking within the composite. As a result, the polymer network becomes more compact, leading to reduced pore size and increased structural integrity. Moreover, higher concentration of particles in the cryogelating solution results in smaller pore sizes.^47,48^ An increase in pore size was observed when comparing CHI-LIG to CHI-LIG-LD, with mode pore diameters of 7.06 and 18.41 μm respectively, which can be attributed to the 2-fold lower monomer concentration in the cryogelation solution used for the preparation of CHI-LIG-LD (Table 1).

**Table 1:** Feed Compositions of the Solutions Used for the Synthesis of QCHI, QCHI-LIG, CHI, CHI-LIG and CHI-LIG-LD

Material	Polymer type	Effective [Polymer] (wt %)	Lignin content (lignin:polymer)	Reduction step
QCHI	Quat. chitosan	4	-	Yes
QCHI-LIG	Quat. chitosan	4	1:1	No
CHI	Chitosan	4	-	Yes
CHI-LIG	Chitosan	4	1:1	No
CHI-LIG-LD	Chitosan	2	1:1	No

After characterizing the cryogel beads, their capacity to adsorb diclofenac from aqueous solutions was evaluated, and the influence of variations in composition and morphology on adsorption efficiency was assessed. Additionally, the performance of the cryogel beads was compared with two commercial materials, AC and IX.

The results of the nonlinear analysis of the data using the pseudo-first order kinetic model and the pseudo-second order kinetic model are summarized in Table 2 and shown in Figure 5a and b, respectively. The rate constants of DF removal by the best-performing cryogel beads, QCHI, were at least 1 order of magnitude higher than those of AC and IX, indicating their superior adsorption performance. Overall, the results obtained with cryogel beads showed a better fit to the pseudo-second order model. During the initial stage of adsorption, the adsorption capacity of the adsorbents for DF increased rapidly, then the process reached equilibrium and q_t_ values reached a constant value. For QCHI, the sorption equilibrium was reached at around 30 min and for QCHI-LIG at around 90 min, while CHI, CHI-LIG, and CHI-LIG-LD reached equilibrium after 240 min. Comparing lignin k_2_ values of QCHI relative to QCHI-LIG and of CHI relative to CHI-LIG, it can be seen that lignin-containing materials have lower adsorption rate constants than their lignin-free counterparts. The beneficial impact of chitosan quaternization on the adsorption kinetics is evident from the higher k_2_ values of QCHI as compared to CHI and of QCHI-LIG compared to CHI-LIG. Moreover, the enhanced porosity of the CHI-LIG-LD beads contributes to an increased k_2_ compared to CHI-LIG, indicating faster adsorption and, consequently, more efficient diclofenac removal within a shorter time frame.

**Figure 5:**
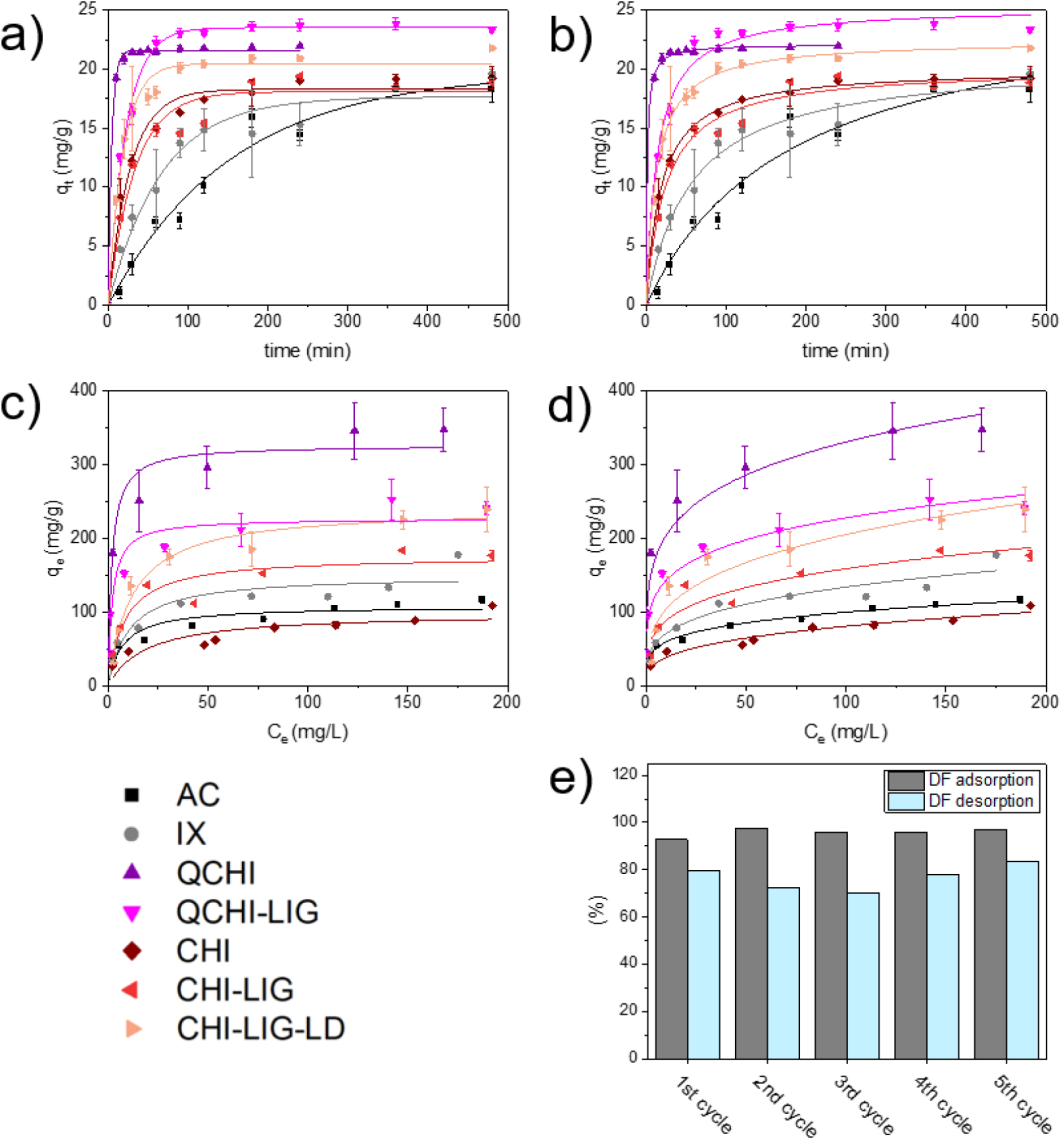
(a) Pseudo-first order model for diclofenac (DF) adsorption on activated carbon (AC), ion-exchange resin (IX), and the five different cryogel beads. b) Pseudo-second order model for DF adsorption on AC, IX, and cryogel beads. c) Langmuir isotherm model for DF adsorption on AC, IX, and cryogel beads. d) Freundlich isotherm model for DF adsorption on AC, IX, and cryogel beads. e) Diclofenac adsorption–desorption performance of QCHI-LIG beads over five cycles. The DF adsorption percentage of QCHI-LIG beads in each cycle is indicated by gray bars. The DF desorption percentage is indicated by blue bars.

**Table 2:** Parameters of Kinetic Models Calculated from Experimental DF Adsorption Data

	Pseudo-first order model		Pseudo-second order model	
Material	k_1_ (1/min)	R^2^	k_2_ (g/(mg min))	R^2^
AC	0.0065	0.9717	0.0002	0.9661
IX	0.0150	0.9511	0.0008	0.9753
QCHI	0.2170	0.9988	0.0333	0.9994
QCHI-LIG	0.0446	0.9940	0.0028	0.9838
CHI	0.0363	0.9732	0.0026	0.9985
CHI-LIG	0.0300	0.9492	0.0021	0.9764
CHI-LIG-LD	0.0531	0.9812	0.0035	0.9937

Adsorption isotherms for DF removal by cryogel beads were investigated and the results of nonlinear analysis of the data using the Langmuir and Freundlich models are summarized in Table 3 and shown in Figure 5c and d, respectively. By fitting the data to the Langmuir model, the maximum adsorption capacity of each material was estimated. CHI exhibited the lowest maximum adsorption capacity (*q*_max_) of all the materials at 99.22 mg/g. The *q*_max_ of CHI-LIG and CHI-LIG-LD were 176.16 mg/g and 241.35 mg/g, respectively, suggesting the beneficial effect of lignin on the DF removal by beads. Moreover, the higher porosity of CHI-LIG-LD corresponded to the highest adsorption capacity among the three nonquaternized materials, highlighting the critical role of the bead’s porous structure in DF removal. QCHI, with a *q*_max_ of 326.55 mg/g, exhibited the highest adsorption capacity for DF among all investigated materials, surpassing QCHI-LIG, which had a *q*_max_ of 227.18 mg/g. These results confirmed the beneficial effect of chitosan quaternization and crucial role of electrostatic interactions on the adsorption efficacy of the cryogel beads for DF removal, which is in agreement with reported data in literature.^36,37^ With the exception of CHI, all cryogel bead adsorbents exhibited higher *q*_max_ values than the tested IX and AC. The K_F_ values of the cryogel beads followed the same trend as the *q*_max_ values, confirming the beneficial effect of chitosan quaternization. The 1/*n* values of the cryogel beads are consistently between 0 and 1, indicating favorable adsorption and interactions between DF and beads. While both the Freundlich and Langmuir isotherm models can describe the isotherm curves of the cryogel adsorbents, the Freundlich model seems to provide a better fit for IX and AC, according to the R^2^ values (Table 3). This suggests that the two commercial adsorbents exhibit adsorption on energetically heterogeneous surfaces, possibly involving multilayer adsorption. In contrast, the cryogel beads appear to exhibit a combination of monolayer and multilayer adsorption on heterogeneous surfaces.^30,49,50^

**Table 3:** Parameters of Langmuir and Freundlich Models Calculated from Experimental DF Adsorption Data

	Langmuir model			Freundlich model		
	*q*_max_ (mg/g)	K_L_ (L/mg)	R^2^	1/*n*	K_F_ ((mg/g)/(mg/L)^1/*n*^)	R^2^
AC	108.33	0.140	0.6984	0.223	35.97	0.9823
IX	150.45	0.100	0.7877	0.274	38.02	0.9166
QCHI	326.55	0.533	0.9504	0.209	126.34	0.9378
QCHI-LIG	227.18	0.487	0.8951	0.201	90.33	0.9408
CHI	99.22	0.053	0.7185	0.318	18.78	0.9313
CHI-LIG	176.16	0.121	0.8601	0.252	49.81	0.8582
CHI-LIG-LD	241.35	0.085	0.9583	0.294	53.02	0.8778

The regenerability of the beads is a crucial factor for their application in water treatment and organic contaminant adsorption, as it can extend the operational lifespan of the adsorbent materials. Several strategies can be used for bead regeneration based on pH,^51,52^ ionic strength,^53^ or different solvent systems.^54,55^ In this study, the modification of the ionic strength was the preferred method of bead regeneration as the use of organic solvents would be undesirable and impractical for applications in (waste)­water treatment plants. In this work, the regeneration of QCHI-LIG was investigated using 0.1 M NaCl as the regeneration solution. QCHI-LIG was chosen as the most suitable candidate for further investigation due to its good adsorption properties and straightforward synthesis procedure, which does not require a reduction step. QCHI-LIG adsorbed 93% of DF in the first cycle, and DF adsorption varied from 95% to 97% over the subsequent four cycles, showing the high regenerability of the beads. DF concentration in the regeneration solution was measured after each cycle. Desorption of diclofenac over 5 cycles ranged from 70% to 83%, indicating the high desorption efficiency of DF from QCHI-LIG. Overall, these results showed that QCHI-LIG has a high potential as a reusable adsorbent for DF removal.

## 4 Conclusion

In this study, five types of chitosan-based cryogel beads were synthesized, characterized, and evaluated regarding their potential as adsorbents for diclofenac removal from water in comparison to two commercially available water treatment materials, one activated carbon and one ion-exchange resin. Five bead types were produced using either native or GTMAC-quaternized chitosan, alone and in combination with lignin, at different polymer concentrations. The macroporous structure of the cryogel beads was investigated using scanning electron microscopy and mercury intrusion porosimetry. The kinetic and equilibrium adsorption behaviors were modeled using the pseudo-first and pseudo-second order models, and Langmuir and Freundlich models, respectively. Quaternization of chitosan increased the adsorption capacity and adsorption rate of cryogel beads toward diclofenac. The incorporation of lignin was shown to slightly reduce diclofenac adsorption kinetics of the cryogel beads but provide a beneficial effect on their stability. Overall, cryogel beads showed superior adsorption performance toward diclofenac compared to an ion-exchange resin and activated carbon. Furthermore, they demonstrated a high regenerability with minimum decrease in their adsorption efficiency over five adsorption–desorption cycles.

This work paves the way for future studies involving actual (waste)­water matrices, flow-through column experiments for long-term performance testing, and the evaluation of the novel materials for the removal of other pollutants from water.

## Supplementary Material





## Notes

Detailed material composition, ^1^H NMR spectra of quaternized chitosan and calculation of the degree of quaternization, physicochemical properties of cryogel beads, initial and final pH values of adsorption isotherm experiments, elemental analysis, calculation of desorption percentages, and photos used for ImageJ analysis (PDF)
^1^H NMR spectra of quaternized chitosan (ZIP)
