# Supplementary material for: Chitosan-Lignin Cryogel Beads for the Adsorption of Diclofenac in Water
Source: Biomacromolecules. 2026 Jul 10;27(8):5212–22. doi: 10.1021/acs.biomac.6c00420 (PMC13463547; doi:10.1021/acs.biomac.6c00420)
Supplement: Supplementary file 1 [file bm6c00420_si_001.pdf]

Supporting Information

Chitosan-lignin cryogel beads for the adsorption of diclofenac in water

Ana Zidar[1]‡, Alejandro Lorente[1]‡, Daniel Kutifa[1], Stephanie Spahr[2], Mohsen Adeli[1,3], Rainer Haag[1], Olaf Wagner[1]**

[1]Institute of Chemistry and Biochemistry, Free University of Berlin, 14195 Berlin, Germany

[2]Deptartment of Ecohydrology and Biogeochemistry, Leibniz Institute of Freshwater Ecology and Inland Fisheries (IGB), 12587 Berlin, Germany

[3]Department of Organic Chemistry, Faculty of Chemistry, Lorestan University, Khorramabad 68141-54316, Iran.

Contents

Detailed feed composition 2

pH values of adsorption isotherm experiment 2

Calculation of desorption experiment results 3

^{1}H NMR and degree of quaternization 5

Physicochemical properties of cryogel beads 6

Elemental analysis 6

ImageJ analysis photos 8

Detailed feed composition

Table S1: Detailed feed composition for each cryogel bead variation.

Material	Polymer type	Polymer mass (g)	Acetic acid volume (mL)	H_2O volume (mL)	GA mass (g)	[GA] (wt%)	GA solution volume	Polymer:GA mass relation	Effective [Polymer] (wt%)	Filler mass (g)	Polymer: Filler mass relation	Reduction step
**QCHI**	Quat. chitosan	4	0	92	0.4	5	8	1:0.1	4	0	-	Yes
**QCHI-LIG**	Quat. chitosan	2	0	46	0.2	5	4	1:0.1	4	2	1:1	No
**CHI**	Chitosan	4	4	88	0.4	5	8	1:0.1	4	0	-	Yes
**CHI-LIG**	Chitosan	2	2	44	0.2	5	4	1:0.1	4	2	1:1	No
**CHI-LIG-LD**	Chitosan	2	2	94	0.2	5	4	1:0.1	2	2	1:1	No

pH values of adsorption isotherm experiment

Table S2: Initial and final pH values of the adsorption isotherm experiments.

		Experiment (final pH value)				
DF concentration (mg/L)	Initial pH value	QCHI	QCHI-LIG	CHI	CHI-LIG	CHI-LIG-LD
10	6.3	6.4	5.2	5.6	5.0	4.6
20	6.3	6.7	5.5	6.0	5.3	4.6
40	6.2	6.8	5.4	6.5	5.5	4.8
70	6.3	7.9	5.7	6.2	6.0	5.3
115	6.3	8.3	5.9	6.6	6.0	5.8
160	6.3	8.7	6.1	6.7	6.2	6.0
200	6.4	8.8	6.0	6.8	6.2	6.0
250	6.3	9.0	6.2	6.7	6.4	6.2

Calculation of desorption experiment results

Equations S1: Calculation of desorption experiment results.

Cryogel beads were loaded with 25 mL of 40 mg/L DF solution (C_0) for 24 h. After 24 h, the DF concentration was measured in the solution to determine the amount of DF adsorbed and the amount of DF that the beads had adsorbed, q_{ads} (mg) was calculated for each cycle:

$$q_{ads} = C_{ads} * 0.025\, L$$

where C_{ads} is the concentration of DF adsorbed.

Percentage of DF adsorbed in each cycle was calculated:

$$\%_{ads} = \frac{q_{ads} * 100}{C_0 * 0.025\, L}$$

where C_0 is the measured initial concentration.

Then the beads were filtered and introduced into 50 mL of 0.1 M NaCl and shaken for 1 h. The DF concentration of the regeneration solution was measured to determine the amount of DF desorbed, which was calculated as:

$$q_{des} = C_{des} * 0.050\, L$$

where C_{des} is the concentration of DF in the desorption solution.

Percentage of DF desorbed in each cycle was calculated for the first cycle as:

$$\%_{des} = \frac{q_{des} * 100}{q_{ads}}$$

and for the following cycles as:

$$\%_{des} = \frac{q_{des} * 100}{q_{ads} + q_r}$$

where q_r is the amount of DF remaining on the beads from the previous cycle. Example calculation for cycle 2:

$$q_{r2} = q_{ads1} - q_{des1}$$

¹H NMR and degree of quaternization

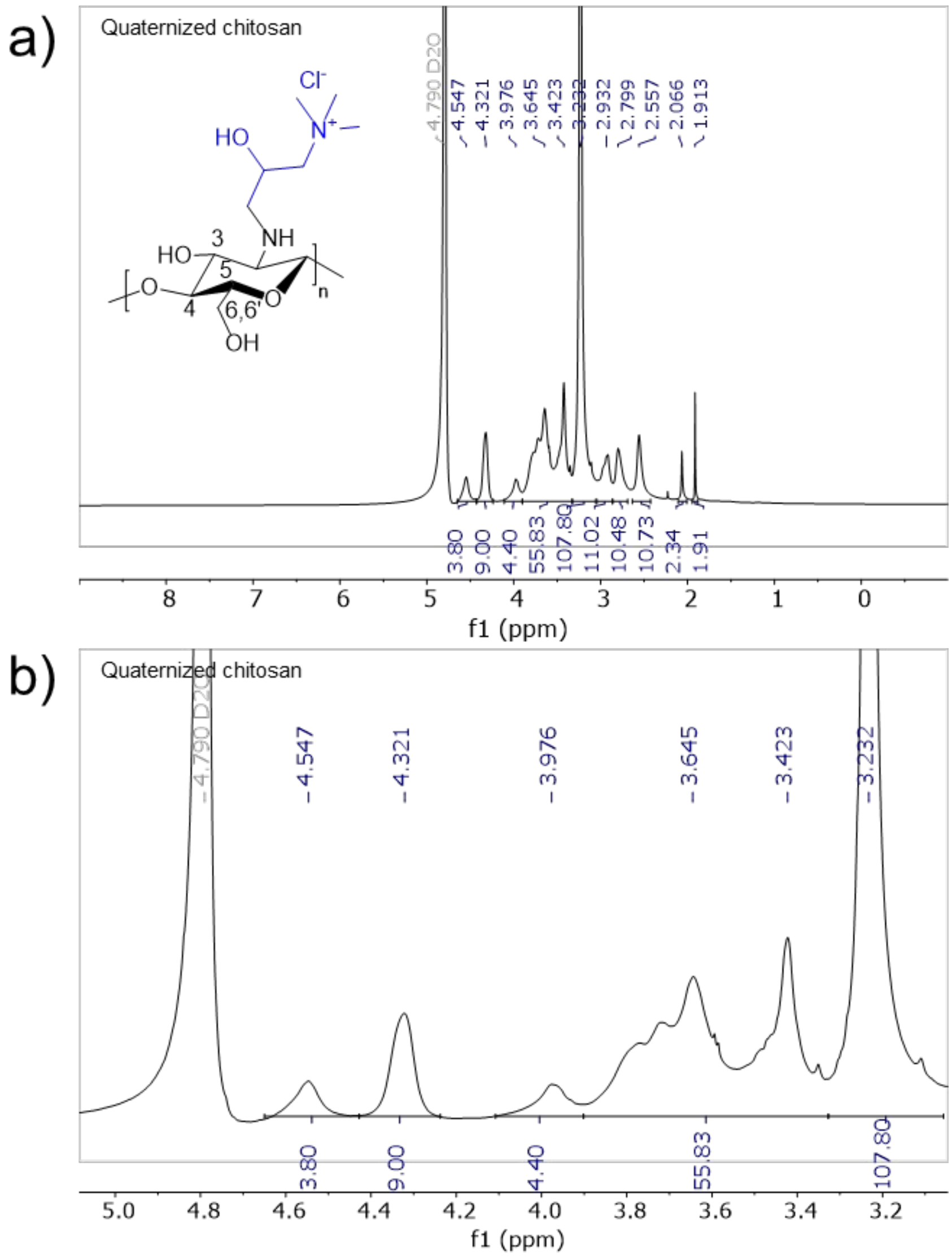

Figure S1. ¹H NMR (600 MHz, D_2O) spectra of quarternized chitosan: a) full spectrum; b) enlarged view.

¹H NMR (600 MHz, D_2O) δ 4.55 (s, 3H), 4.32 (s, 8H), 3.98 (s, 1H), 3.90 – 3.33 (m, 12H), 3.23 (s, 107H), 2.93 (s, 1H), 2.80 (s, 1H), 2.56 (s, 6H), 2.07 (s, 1H), 1.91 (s, 1H).

The degree of quaternization (DQ) can be determined by the integral ratio of protons in the $N(CH_3)_3$ group to the protons $H_{3\text{-}6}$ on the chitosan backbone according to Equation S2.

The protons on the quaternary ammonium group correspond to a singlet peak at 3.232 ppm, while the H_3, H_4, H_5, H_6, and $H_{6'}$ protons correspond to the 4 peaks between 3.3 and 4.4 ppm.

Equation S2: $DQ = (I_{N(Me)3}/9) / (I_{H3\text{-}H6}/5) * 100 = (107.80/9) / (69.23/5) *100 = 86.51\%$

The degree of quaternization of the quaternized chitosan used in this study was 86.51%.

Physicochemical properties of cryogel beads

Table S3. Physicochemical properties of cryogel beads with different compositions.

Material	Swelling degree (%)	Density (mg/mL)[1]	Bulk density (mg/mL)[2]	Mode pore diameter (µm) [2]	Median bead diameter (mm)[3]
QCHI	2170	46.61	38.1	45.20	4.27±0.88
QCHI-LIG	2151	88.16	87.8	14.72	5.12±0.81
CHI	1762	72.94	57.9	11.76	3.85±0.55
CHI-LIG	1033	109.55	110.0	7.06	4.25±0.71
CHI-LIG-LD	1842	37.73	72.9	18.41	6.69±1.79

[1] calculated from bead weight and diameter

[2] determined with MIP

[3] determined from SEM images using ImageJ software

Elemental analysis

Table S4: Elemental analysis

	Elemental analysis (%)			
Material	N	C	S	H
Chitosan	6.903	40.33	0.005	6.627

Lignin	0.084	46.73	0.000	6.612
Quat. chitosan	8.117	46.90	0.000	9.784
QCHI	7.618	45.50	0.000	10.61
QCHI-LIG	3.132	45.38	0.000	7.561
CHI	6.850	42.28	0.000	6.937
CHI-LIG	3.240	51.28	0.783	6.247
CHI-LIG-LD	2.723	47.24	0.000	7.148

Elemental analysis was used to verify the success of the addition of quaternary ammonium groups on chitosan and the addition of lignin in the materials. To confirm the successful conjugation of GTMAC to chitosan we can compare the nitrogen content of quaternized chitosan with non-modified chitosan. The nitrogen content of quaternized chitosan is higher (8.117%) in comparison with chitosan (6.903%), which confirms the addition of quaternary ammonium groups on the chitosan.

ImageJ analysis photos

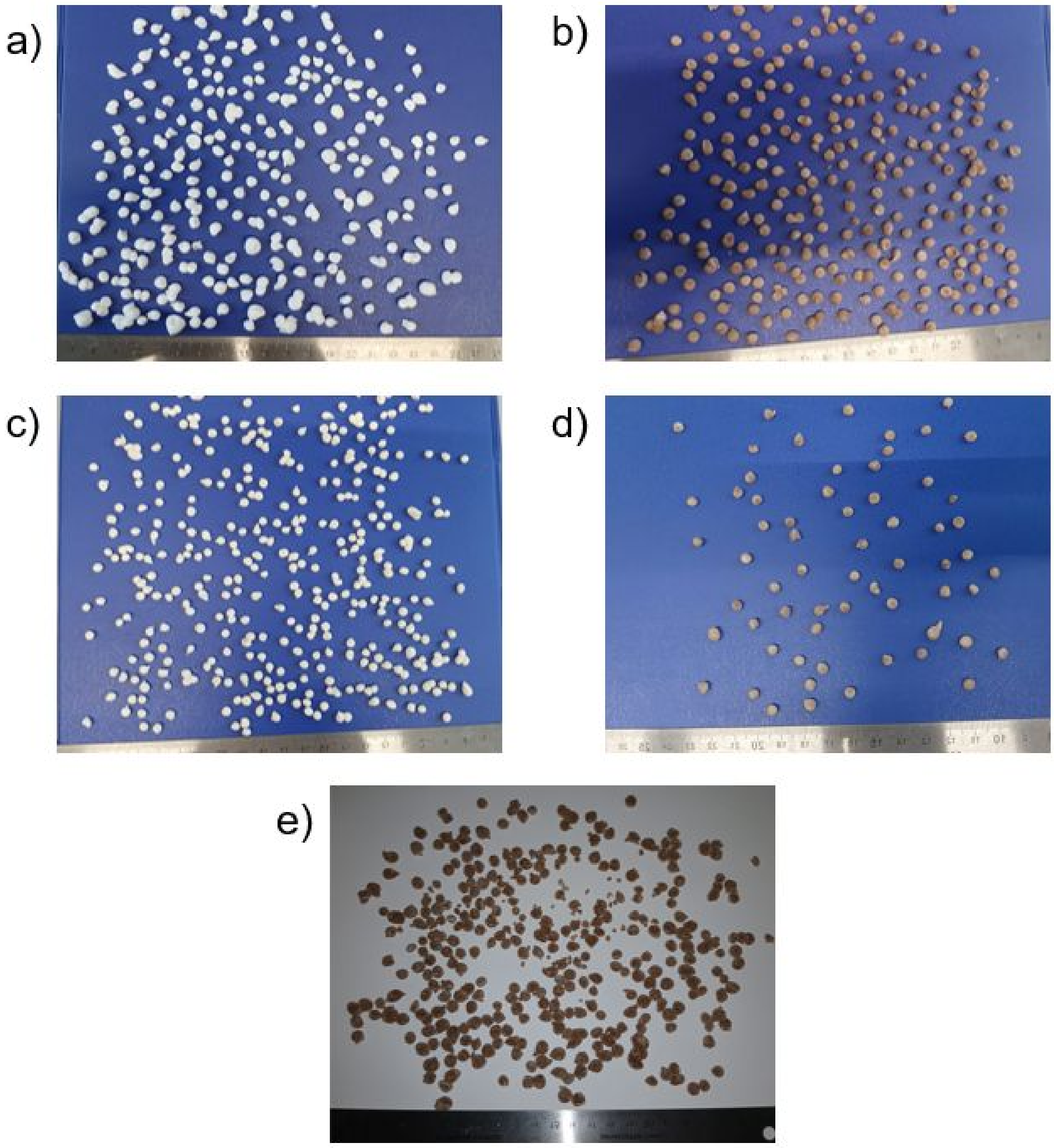

Figure S2: images used for ImageJ analysis for determination of bead diameter: a) QCHI, b) QCHI-LIG, c) CHI, d) CHI-LIG, e) CHI-LIG-LD.
